# Existence and global exponential stability of periodic solutions for *n*-dimensional neutral dynamic equations on time scales

**DOI:** 10.1186/s40064-016-1872-7

**Published:** 2016-02-29

**Authors:** Bing Li, Yongkun Li, Xuemei Zhang

**Affiliations:** Department of Mathematics, Yunnan University, Kunming, 650091 Yunnan People’s Republic of China; School of Mathamatics and Information Science, Qujing Normal University, Qujing, 655011 Yunnan People’s Republic of China

**Keywords:** Periodic solution, Neutral delay, Exponential stability, *n*-Dimensional neutral dynamic equations, Time scale, 34K13, 34K20, 34K40, 34N05

## Abstract

In this paper, by using the existence of the exponential dichotomy of linear dynamic equations on time scales and the theory of calculus on time scales, we study the existence and global exponential stability of periodic solutions for a class of *n*-dimensional neutral dynamic equations on time scales. We also present an example to illustrate the feasibility of our results. The results of this paper are completely new and complementary to the previously known results even in both the case of differential equations (time scale ${\mathbb {T}}={\mathbb {R}}$) and the case of difference equations (time scale ${\mathbb {T}}={\mathbb {Z}}$).

## Background

The theory of dynamic equations on time scales was introduced by Hilger (1990) in 1988 in order to unify the study of continuous and discrete calculus. Since then, the study on dynamic equations on time scales has received much attention of many scholars. For example, in DaCunha (2005), the author studied the stability of the following linear dynamic equation on time scales:

$$x^\Delta (t)=A(t)x(t),\; x(t_0)=x_0,\quad t_0\in {\mathbb {T}}.$$

In Du and Tien (2007), the authors obtained some conditions ensuring the stability of the trivial solution for the following dynamic equation on time scales:

$$x^\Delta (t)=A(t)x(t)+f(t,x),\quad t\in {\mathbb {T}}.$$

For other studies on dynamic equations on time scales, we refer the reader to Bohner and Peterson (2001), Graef and Hill (2015), Li and Sun (2013), Li and Xu (2011), Lupulescu and Younus (2011), Su and Feng (2014), Wang et al. (2010), Zhang et al. (2010a, b, 2014), Zhou and Li (2010, 2012) and the references therein.

Since it is natural and important that systems will contain some information about the derivative of the past state to further describe and model the dynamics for such complex reactions, many authors have studied the existence of solutions of various neutral delay models (Abbas and Bahuguna 2008; Ardjouni and Djoudi 2012; Chen and Lin 2010; Hovhannisyan 2014; Kaufmann and Raffoul 2006; Li and Saker 2014; Xu et al. 2007; Zhang et al. 2009). However, to the best of our knowledge, there are few papers published on the existence and stability of periodic solutions to neutral dynamic equations on time scales.

Motivated by the above discussion, in this paper, we are concerned with the following neutral dynamic equation on time scales:

1 $$x^\Delta (t)=A(t)x(t)+f\left( t,x_t,x^\Delta _t\right) ,\quad t\in {\mathbb {T}},$$

where ${\mathbb {T}}$ is an $\omega$-periodic time scale and satisfies that for $t,s\in {\mathbb {T}}, t+s\in {\mathbb {T}}$, $A(t)=(a_{ij}(t))_{n\times n}$ is a regressive and rd-continuous matrix-valued function, $f\in C_{rd}({\mathbb {T}}\times BC\times BC, {\mathbb {R}}^n)$ and $f(t,x_{t},x^\Delta _t)$ is $\omega$-periodic whenever *x* is a $\Delta$-differentiable $\omega$-periodic function with rd-continuous $\Delta$-derivative, where *BC* denotes the Banach space of all bounded rd-continuous functions $\varphi : [-\theta ,0]\cap {\mathbb {T}}\rightarrow {\mathbb {R}}^{n}$ with the norm $|\varphi |_0= \max \nolimits _{1\le i\le n}\sup \nolimits _{s\in [-\theta ,0]\cap {\mathbb {T}}}|\varphi _{i}(s)|$ where $\varphi =(\varphi _{1},\phi _{2},\ldots ,\varphi _{n})^{T}, \omega > 0$ is a constant, $\theta$ is a positive number or $\infty$ and if $\theta =\infty$, then we set $[-\theta ,0]= (-\infty ,0]$. If $x,x^\Delta \in C_{rd}({\mathbb {T}},{\mathbb {R}}^n)$, then for any $t\in {\mathbb {T}}$, $x_{t}$ and $x^\Delta _t\in BC$ are defined by $x_{t}(s)=x(t+s)$ and $x^\Delta _{t}(s)=x^\Delta (t+s)$ for $s\in [-\theta ,0]\cap {\mathbb {T}}$, respectively.

### *Remark 1*

Throughout this paper, we denote the class of all functions $f: {\mathbb {T}}\times BC\times BC\rightarrow {\mathbb {R}}^n$ that are rd-continuous with respect to their first argument and continuous with respect to their second and third arguments by $C_{rd}({\mathbb {T}}\times BC\times BC, {\mathbb {R}}^n)$.

### *Remark 2*

If $\theta$ is a finite positive number, then Eq. (1) is a bounded delay neutral dynamic equation on time scales and if $\theta$ is infinite then Eq. (1) is a unbounded delay neutral dynamic equation on time scales.

Our main purpose of this paper is to study the existence and global exponential stability of periodic solutions for (1) by using the exponential dichotomy of linear dynamic equations and the theory of calculus on time scales. As we all know, Eq. (1) contains many differential equation models and difference equation models as its special cases. For example, if we take

$$\begin{aligned} {\mathbb {T}}={\mathbb {R}}, \quad A(t) &= {\mathrm {diag}}[-b_{1}(t),-b_{2}(t),\ldots ,-b_{n}(t)],\quad f=(f_1,f_2,\ldots ,f_n),\\ f_i(t,\varphi ,\varphi ') &= \sum \limits _{j=1}^na_{ij}(t)f_j(\varphi _j(0))+ \sum \limits _{j=1}^nb_{ij}(t)f_j(\varphi _j(-\tau _{ij}(t)))\\&\quad + c_{i}\varphi _i'(-\sigma _{i}(t))+I_i(t),\quad i=1,2,\ldots ,n, \end{aligned}$$

where $\varphi =(\varphi _1,\varphi _2,\ldots ,\varphi _n)$, then (1) reduces to the following neural network with neutral type delays:

$$\begin{aligned} x_i'(t) &= -b_i(t)x_i(t)+\sum \limits _{j=1}^na_{ij}(t)f_j(x_j(t))+ \sum \limits _{j=1}^nb_{ij}(t)f_j(x_j(t-\tau _{ij}(t)))\\&\quad +c_{i}x_i'(t-\sigma _{i}(t))+I_i(t),\quad i=1,2,\ldots ,n, \end{aligned}$$

which was studied in Li et al. (2012). If we take

$$\begin{aligned}&{\mathbb {T}}={\mathbb {R}}, \quad A(t)={\mathrm {diag}}[a_{1}(t),a_{2}(t),\ldots ,a_{n}(t)],\\ f(t,\varphi ,\varphi ')= \lambda F(t,\varphi _t), \end{aligned}$$

then (1) reduces to

$$x'=A(t)x(t)+\lambda F(t,x_t),$$

which was studied in Liu and Li (2004). Even in both the case of differential equations (time scale ${\mathbb {T}}={\mathbb {R}})$ and the case of difference equations $({\mathbb {T}}={\mathbb {Z}})$, our results are completely new and complementary to the previously known results.

For convenience, we denote $$[a,b]_{\mathbb {T}}=\{t| t\in [a, b]\cap {\mathbb {T}}\}$$. For an rd-continuous $$\omega$$-periodic function $$h:{\mathbb {T}}\rightarrow {\mathbb {R}}$$, denote $$h^+=\sup \nolimits _{t\in [0,\omega ]_{\mathbb {T}}}|h(t)|, h^-=\inf \nolimits _{t\in [0,\omega ]_{\mathbb {T}}}|h(t)|$$. For an rd-continuous $$\omega$$-periodic function $$u:{\mathbb {T}}\rightarrow {\mathbb {R}}^n$$, we define $$|u|_0 =\max \nolimits _{1\le i\le n}\max \nolimits _{t\in [0,\omega ]_{\mathbb {T}}}|u_i(t)|$$. For matrices or vectors *A*, *B*, $$A\ge B$$ (or $$A>B)$$ means that all entries of *A* are greater than or equal to (or greater than) corresponding entries of *B*. For $$A(t)=(a_{ij}(t))_{n\times n}$$, we can take $$||A||=\max \nolimits _{1\le i\le n}\sum \nolimits _{j=1}^n|a_{ij}^+|$$.

The initial condition of (1) is

$$x(s)=\phi (s),\; x^\Delta (s)=\phi ^\Delta (s),\quad s\in [-\theta ,0]_{{\mathbb {T}}},$$

where $$\phi \in C^1_{rd}([-\theta ,0]_{{\mathbb {T}}}, {\mathbb {R}}^n)$$.

Throughout this paper, we assume that the following condition holds:

$$(H_{1})$$
$f\in C_{rd}({\mathbb {T}}\times BC\times BC,{\mathbb {R}}^n)$ is $\omega$-periodic with respect to its first argument and there exist positive constants $L_1, L_2$ such that
  $$|f(t,\varphi _1, \psi _1)-f(t,\varphi _2,\psi _2)|_0\le L_1|\varphi _1-\varphi _2|_0+L_2|\psi _1-\psi _2|_0$$
  for all $t\in {\mathbb {T}}$ and $\varphi _i, \psi _i\in BC, i=1,2$.

## Preliminaries

In this section, we introduce some definitions and state some preliminary results.

Let ${\mathbb {T}}$ be a nonempty closed subset (time scale) of ${\mathbb {R}}$. The forward and backward jump operators $\sigma , \rho :{\mathbb {T}}\rightarrow {\mathbb {T}}$ and the graininess $\mu :{\mathbb {T}}\rightarrow {\mathbb {R}}^+$ are defined, respectively, by

$$\sigma (t)=\inf \{s\in {\mathbb {T}}:s>t\},\quad \rho (t)=\sup \{s\in {\mathbb {T}}:s<t\}\quad \mathrm{and}\quad \mu (t)=\sigma (t)-t.$$

A point $t\in {\mathbb {T}}$ is called left-dense if $t>\inf {\mathbb {T}}$ and $\rho (t)=t$, left-scattered if $\rho (t)<t$, right-dense if $t<\sup {\mathbb {T}}$ and $\sigma (t)=t$, and right-scattered if $\sigma (t)>t$. If ${\mathbb {T}}$ has a left-scattered maximum *m*, then ${\mathbb {T}}^k={\mathbb {T}}{\setminus} \{m\}$; otherwise ${\mathbb {T}}^k={\mathbb {T}}$. If ${\mathbb {T}}$ has a right-scattered minimum *m*, then ${\mathbb {T}}_k={\mathbb {T}}{\setminus} \{m\}$; otherwise ${\mathbb {T}}_k={\mathbb {T}}$.

A function $f:{\mathbb {T}}\rightarrow {\mathbb {R}}$ is right-dense continuous provided it is continuous at right-dense points in ${\mathbb {T}}$ and its left-side limits exist at left-dense points in ${\mathbb {T}}$. If *f* is continuous at each right-dense point and each left-dense point, then *f* is said to be continuous on ${\mathbb {T}}$. We denote the class of all rd-continuous functions $f:{\mathbb {T}}\rightarrow {\mathbb {R}}$ by $C_{rd}({\mathbb {T}},{\mathbb {R}})$.

For $y:{\mathbb {T}}\rightarrow {\mathbb {R}}$ and $t\in {\mathbb {T}}^k$, we define the delta derivative of *y*(*t*), $y^\Delta (t)$, to be the number (if it exists) with the property that for a given $\varepsilon >0$, there exists a neighborhood *U* of *t* such that

$$|[y(\sigma (t))-y(s)]-y^\Delta (t)[\sigma (t)-s]|<\varepsilon |\sigma (t)-s|$$

for all $$s\in U$$.

We denote the class of all $\Delta$-differentiable functions with rd-continuous $\Delta$-derivative $f:{\mathbb {T}}\rightarrow {\mathbb {R}}$ by $C_{rd}^1({\mathbb {T}},{\mathbb {R}})$.

If *y* is continuous, then *y* is right-dense continuous, and if *y* is $\Delta$-differentiable at *t*, then *y* is continuous at *t*.

Let *y* be right-dense continuous. If $Y^{\Delta }(t)=y(t)$, then we define the delta integral by $\int _a^{t}y(s)\Delta s=Y(t)-Y(a)$. Assume that $f: {\mathbb {T}}\rightarrow {\mathbb {R}}^n$ is a function and $f(t)=(f_1(t),\ldots ,f_n(t))$, then we define $\int _a^{t}f(s) \Delta s=(\int _a^{t}f_1(s)\Delta s,\ldots ,\int _a^{t}f_n(s)\Delta s)$(provided it exists).

### **Definition 1**

(Bohner and Peterson 2001) Let *A* be an $m\times n$-matrix-valued function on ${\mathbb {T}}$. We say that *A* is *rd*-continuous on ${\mathbb {T}}$ if each entry of *A* is *rd*-continuous on ${\mathbb {T}}$. We say that *A* is differentiable on ${\mathbb {T}}$ provided each entry of *A* is differentiable on ${\mathbb {T}}$, and in this case we put $A^\Delta =(a_{ij}^\Delta )_{m\times n}$, where $A=(a_{ij})_{m\times n}$.

### **Definition 2**

(Kaufmann and Raffoul 2006) We say that a time scale ${\mathbb {T}}$ is periodic if there exists $p>0$ such that if $t\in {\mathbb {T}}$, then $t\pm p\in {\mathbb {T}}$. For ${\mathbb {T}}\ne \,{\mathbb {R}}$, the smallest positive *p* is called the period of the time scale.

### **Definition 3**

(Kaufmann and Raffoul 2006) Let ${\mathbb {T}}\ne {\mathbb {R}}$ be a periodic time scale with period *p*. We say that the function $f:{\mathbb {T}}\rightarrow {\mathbb {R}}$ is periodic with period $\omega$ if there exists a natural number *n* such that $\omega =np$, $f(t+\omega )=f(t)$ for all $t\in {\mathbb {T}}$ and $\omega$ is the smallest positive number such that $f(t+\omega )=f(t)$.

### **Definition 4**

(Bohner and Peterson 2001) A $n\times n$-matrix-valued function *A* on time scale ${\mathbb {T}}$ is called regressive (respect to ${\mathbb {T}}$) provided $I+\mu (t)A(t)$ is invertible for all $t\in {\mathbb {T}}^k$.

### **Definition 5**

(Bohner and Peterson 2001) Let *A*, *B* be two $n\times n$-matrix-valued regressive functions on ${\mathbb {T}}$, we define

$$\begin{aligned}&(A\oplus B)(t):=A(t)+B(t)+\mu (t)A(t)B(t),\\&(\ominus A)(t):=-[I+\mu (t)A(t)]^{-1}A(t)=-A(t)[I+\mu (t)A(t)]^{-1},\\&(A(t))\ominus (B(t)):=(A(t))\oplus (\ominus (B(t))) \end{aligned}$$

for all $$t\in {\mathbb {T}}^k$$.

### **Definition 6**

(Bohner and Peterson 2001) Let $t_0\in {\mathbb {T}}$ and assume that $A\in {\mathcal {R}}$ is a $n\times n$-matrix-valued function. The unique matrix-valued solution of the initial value problem

$$x^{\Delta }(t)=A(t)x(t),\quad x(t_0)=I,$$

where, *I* denotes as usual the $n\times n$-identity matrix, is called the matrix exponential function (at $t_0$) and it is denoted by $e_A(\cdot ,t_0)$.

### *Remark 3*

Assume that *A* is a constant $n\times n$-matrix. If ${\mathbb {T}}={\mathbb {R}}$, then $e_A(t,t_0)=e^{A(t-t_0)}$, while if ${\mathbb {T}}={\mathbb {Z}}$ and $I+A$ is invertible, then $e_A(t,t_0)=(I+A)^{(t-t_0)}$.

### **Lemma 1**

(Bohner and Peterson 2001) *Let*$A\in {\mathcal {R}}$*be a*$n\times n$-*matrix-valued functions on*${\mathbb {T}}$*and suppose that*$f:{\mathbb {T}}\rightarrow {\mathbb {R}}^n$*isrd*-*continuous. Let*$t_0\in {\mathbb {T}}$*and*$x_0\in {\mathbb {R}}^n$. *Then the initial value problem*

$$x^{\Delta }(t)=A(t)x(t)+f(t),\quad x(t_0)=x_0$$

*has a unique solution*$x:{\mathbb {T}}\rightarrow {\mathbb {R}}^n$, *which is given by*

$$x(t)=e_A(t,t_0)x_0+\int _{t_0}^te_A(t,\sigma (s))f(s)\Delta s.$$

### **Lemma 2**

(Bohner and Peterson 2001) *If*$A,B\in {\mathcal {R}}$*are matrix-valued functions on*${\mathbb {T}}$, *then*

(i) $$e_0(t,s)\equiv I$$*and*$$e_A(t,t)\equiv I$$;
(ii) $$e_A(\sigma (t),s)=(I+\mu (t)A(t))e_A(t,s)$$;
(iii) $$e_A(t,s)=e_A^{-1}(s,t)$$;
(iv) $$e_A(t,s)e_A(s,r)=e_A(t,r)$$;
(v) $$e_A(t,s)e_B(t,s)=e_{A\oplus B}(t,s)$$, $${\mathrm {if}}$$$$e_A(t,s)$$$${\mathrm {and}}$$*B*(*t*) $${\mathrm {commute}}$$.

### **Lemma 3**

(Bohner and Peterson 2001) *If*$A\in {\mathcal {R}}$*and*$a,b,c\in {\mathbb {T}}$, *then*

$${[}e_A(c,\cdot )]^{\Delta }=-[e_A(c,\cdot )]^{\sigma }A$$

*and*

$$\int _a^{b}e_A(c,\sigma (t))A(t)\Delta t=e_A(c,a)-e_A(c,b).$$

### **Definition 7**

(Zhang et al. 2010a) *Let*$x\in {\mathbb {R}}^{n}$*andA*(*t*) *be a*$n\times n$*matrix-valued function on*${\mathbb {T}}$, *the linear system*

2 $$x^{\Delta }(t)=A(t)x(t),\quad t\in {\mathbb {T}}$$

*is said to admit an exponential dichotomy on*${\mathbb {T}}$*if there exist positive constants*$k_i,\alpha _i,i=1,2$, *projectionPand the fundamental solution matrixX*(*t*) *of* (2) *satisfying*

$$\begin{aligned} &&|X(t)PX^{-1}(s)|_0 \le k_1e_{\ominus \alpha _1}(t,s),\quad s,t\in {\mathbb {T}},\; t\ge s,\\ &&|X(t)(I-P)X^{-1}(s)|_0 \le k_2e_{\ominus \alpha _2}(s,t),\quad s,t\in {\mathbb {T}},\; t\le s. \end{aligned}$$

### **Lemma 4**

(Zhang et al. 2010a) *If* (2) *admits an exponential dichotomy, then the following*$\omega$-*periodic system*:

$$X^{\Delta }(t)=A(t)X(t)+g(t),\quad t\in {\mathbb {T}}$$

*has an*$\omega$-*periodic solution as follows*:

$$X(t)=\int _{-\infty }^{t}X(t)PX^{-1}(\sigma (s))g(s)\Delta s-\int ^{+\infty }_{t}X(t)(I-P)X^{-1}(\sigma (s))g(s)\Delta s,$$

*whereX*(*t*) *is the fundamental solution matrix of* (2).

### **Lemma 5**

(Zhang et al. 2010a) *IfA*(*t*) *is a uniformly boundedrd*-*continuous*$n\times n$*matrix-valued function on*${\mathbb {T}}$*and there is a*$\delta >0$*such that*

$$|a_{ii}(t)|-\sum \limits _{j\ne i}|a_{ij}(t)|-\frac{1}{2}\mu (t)\left( \sum \limits _{j=1}^n|a_{ij}(t)|\right) ^2-\delta ^2\mu (t)\ge 2\delta ,\quad t\in {\mathbb {T}},\; i=1,2,\ldots ,n,$$

*then* (2) *admits an exponential dichotomy on*${\mathbb {T}}$.

### **Definition 8**

Let *x*(*t*) be an $\omega$-periodic solution of (1) with initial value $\varphi (s)$. If there exists a positive constant $\lambda$ with $-\lambda \in {\mathcal {R}}^{+}$ such that for $t_0\in [-\theta ,0]_{{\mathbb {T}}}$, there exists $M>1$ such that for an arbitrary solution *y*(*t*) of (1) with initial value $\psi (s)$ satisfies

$$||y-x||_{\mathbb {X}}\le M||\varphi -\psi ||_{\mathbb {X}}e_{\ominus \lambda }(t,t_0),\quad t\in [-\theta ,\infty )_{{\mathbb {T}}},\; t\ge t_0.$$

Then the solution *x*(*t*) is said to be globally exponentially stable.

## Existence of periodic solutions

Set ${\mathbb {X}}=\{\varphi \in C^1_{rd}({\mathbb {T}},{\mathbb {R}}^n)| \varphi$ is $\omega$-periodic on ${\mathbb {T}}\}$ with the norm $||\varphi ||_{{\mathbb {X}}}=\max \{|\varphi |_0, |\varphi ^\Delta |_0\}$, where $|\varphi |_0 =\max \nolimits _{1\le i\le n}\sup \nolimits _{t\in [0,\omega ]_{\mathbb {T}}}|\varphi _i(t)|$, $|\varphi ^\Delta |_0 =\max \nolimits _{1\le i\le n}\sup \nolimits _{t\in [0,\omega ]_{\mathbb {T}}}|\varphi _i^\Delta (t)|$, then ${\mathbb {X}}$ is a Banach space.

### **Theorem 1**

*Let*$(H_1)$*hold. Suppose that*

$$(H_2)$$
*system* (2) *admits an exponential dichotomy on*${\mathbb {T}}$*with constants*$k_i, \alpha _i$, $i=1,2$;
$$(H_3)$$
$$q=:\max \left\{ \displaystyle \frac{k_1(1+\vartheta \alpha _1)}{\alpha _1} +\frac{k_2}{\alpha _2},||A||\left( \frac{k_1(1+\vartheta \alpha _1)}{\alpha _1} +\frac{k_2}{\alpha _2}\right) +1\right\} (L_1+L_2)<1$$, where $$\vartheta =\sup \nolimits _{t\in {\mathbb {T}}}\mu (t)$$.

*Then* (1) *has a unique*$\omega$-*periodic solution*.

### *Proof*

By $(H_3)$, we can take a positive constant *L* satisfying

$$\begin{aligned} \max \left\{ \displaystyle \frac{k_1(1+\vartheta \alpha _1)}{\alpha _1} +\frac{k_2}{\alpha _2},||A||\left( \frac{k_1(1+\vartheta \alpha _1)}{\alpha _1} +\frac{k_2}{\alpha _2}\right) +1\right\} \left( (L_1+L_2)L+a\right) \le L, \end{aligned}$$

where $a=|f(\cdot ,0,0)|_0$. We set ${\mathbb {X}}_0=\left\{ \varphi \in {\mathbb {X}} |\, ||\varphi ||_{\mathbb {X}}\le L\right\}$. For any given $\varphi \in {\mathbb {X}}_0$, we consider the following periodic system:

3 $$x^\Delta (t)=A(t)x(t) +f\left( t,\varphi _t,\varphi ^\Delta _t\right) .$$

Since $(H_2)$ holds, by Lemma 4, we obtain that (3) has an $\omega$-periodic solution, which is expressed as follows:

$$\begin{aligned} x^\varphi (t)= \int _{-\infty }^tX(t)PX^{-1}(\sigma (s))f\left( s,\varphi _s, \varphi ^\Delta _s\right) \Delta s-\int ^{+\infty }_{t}X(t)(I-P)X^{-1}(\sigma (s))f\left( s,\varphi _s, \varphi ^\Delta _s\right) \Delta s. \end{aligned}$$

For $\varphi \in {\mathbb {X}}_0$, define the following operator:

$$\Phi : {\mathbb {X}}_0\rightarrow {\mathbb {X}}_0, \;\varphi \rightarrow x^\varphi .$$

First we show that for any $\varphi \in {\mathbb {X}}_0$, we have $\Phi \varphi \in {\mathbb {X}}_0$. Note that

$$\begin{aligned} |f\left( s,\varphi _s,\varphi ^\Delta _s\right) |_0 &\le |f\left( s,\varphi _s,\varphi ^\Delta _s\right) -f(s,0,0)|_0+|f(s,0,0)|_0\\&\le L_1|\varphi |_0+L_2|\varphi ^\Delta |_0+a\\ &\le (L_1+L_2)\Vert \varphi \Vert _{\mathbb {X}}+a. \end{aligned}$$

So, we have that

$$\begin{aligned} |\Phi \varphi |_0 &= \left| \int _{-\infty }^tX(t)PX^{-1}(\sigma (s))f\left( s,\varphi _s, \varphi ^\Delta _s\right) \Delta s\right. \\&\quad\left. -\int ^{+\infty }_{t}X(t)(I-P)X^{-1}(\sigma (s))f\left( s,\varphi _s, \varphi ^\Delta _s\right) \Delta s\right| _0\\ &\le \sup \limits _{t\in [0,\omega ]_{\mathbb {T}}}\left( \int _{-\infty }^t\left| X(t)PX^{-1}(\sigma (s))\right| _0\left| f\left( s,\varphi _s, \varphi ^\Delta _s\right) \right| _0\Delta s\right. \\&\quad\left. +\int ^{+\infty }_{t}\left| X(t)(I-P)X^{-1}(\sigma (s))\right| _0\left| f\left( s,\varphi _s, \varphi ^\Delta _s\right) \right| _0\Delta s\right) \\&\le \left( (L_1+L_2)\Vert \varphi \Vert _{\mathbb {X}}+a\right) \left( \sup \limits _{t\in [0,\omega ]_{\mathbb {T}}}\left| \int _{-\infty }^tk_1e_{\ominus \alpha _1}(t,\sigma (s))\Delta s\right| \right. \\&\quad\left. +\sup \limits _{t\in [0,\omega ]_{\mathbb {T}}}\left| \int ^{+\infty }_{t}k_2e_{\ominus \alpha _2}(\sigma (s),t)\Delta s\right| \right) \\ &= \left( (L_1+L_2)\Vert \varphi \Vert _{\mathbb {X}}+a\right) \left( \sup \limits _{t\in [0,\omega ]_{\mathbb {T}}}\left| k_1\int _{-\infty }^t(1+\mu (s)\alpha _1)e_{\alpha _1}(s,t)\Delta s\right| \right. \\& \quad\left. +\sup \limits _{t\in [0,\omega ]_{\mathbb {T}}} \left| k_2\int ^{+\infty }_{t}(1+\mu (s)\ominus \alpha _2)e_{\ominus \alpha _2}(s,t)\Delta s\right| \right) \\ &= \left( (L_1+L_2)\Vert \varphi \Vert _{\mathbb {X}}+a\right) \left( \frac{k_1(1+\vartheta \alpha _1)}{\alpha _1} \sup \limits _{t\in [0,\omega ]_{\mathbb {T}}}\left| \int _{-\infty }^t\alpha _1e_{\alpha _1}(s,t)\Delta s\right| \right. \\&\quad\left. +\sup \limits _{t\in [0,\omega ]_{\mathbb {T}}}\left| -\frac{k_2}{\alpha _2}\int ^{+\infty }_{t}\ominus \alpha _2e_{\ominus \alpha _2}(s,t)\Delta s\right| \right) \\&\le \left( (L_1+L_2)\Vert \varphi \Vert _{\mathbb {X}}+a\right) \left( \frac{k_1(1+\vartheta \alpha _1)}{\alpha _1} +\frac{k_2}{\alpha _2}\right) . \end{aligned}$$

On the other hand, we have

$$\begin{aligned} |(\Phi \varphi )^\Delta |_0 &= \left| \left( \int _{-\infty }^tX(t)PX^{-1}(\sigma (s))f\left( s,\varphi _s, \varphi ^\Delta _s\right) \Delta s\right. \right. \\&\quad\left. \left. -\int ^{+\infty }_{t}X(t)(I-P)X^{-1}(\sigma (s))f\left( s,\varphi _s, \varphi ^\Delta _s\right) \Delta s\right) ^\Delta _t\right| _0\\ &= \left| f\left( t,\varphi _s, \varphi ^\Delta _s\right) +A(t)\left( \int _{-\infty }^tX(t)PX^{-1}(\sigma (s))f\left( s,\varphi _s, \varphi ^\Delta _s\right) \Delta s\right. \right. \\&\quad\left. \left. -\int ^{+\infty }_{t}X(t)(I-P)X^{-1}(\sigma (s))f\left( s,\varphi _s, \varphi ^\Delta _s\right) \Delta s\right) \right| _0\\&\le \left( (L_1+L_2)\Vert \varphi \Vert _{\mathbb {X}} +a\right) \left( ||A||\left( \frac{k_1(1+\vartheta \alpha _1)}{\alpha _1} +\frac{k_2}{\alpha _2}\right) +1\right) . \end{aligned}$$

Hence, we have $||\Phi \varphi ||_{\mathbb {X}}\le L$, that is, $\Phi \varphi \in {\mathbb {X}}_0$. Next, we show that $\Phi$ is a contraction. For any $\varphi ,\psi \in {\mathbb {X}}_0$, we have

$$\begin{aligned} |\Phi \varphi -\Phi \psi |_0 &= \left| \int _{-\infty }^tX(t)PX^{-1}(\sigma (s))\left( f\left( s,\varphi _s, \varphi ^\Delta _s\right) -f\left( s,\psi _s, \psi ^\Delta _s\right) \right) \Delta s\right. \\&\quad\left. -\int ^{+\infty }_{t}X(t)(I-P)X^{-1}(\sigma (s))\left( f\left( s,\varphi _s, \varphi ^\Delta _s\right) -f\left( s,\psi _s, \psi ^\Delta _s\right) \right) \Delta s\right| _0\\ &\le \sup \limits _{t\in [0,\omega ]_{\mathbb {T}}}\left| \int _{-\infty }^t|X(t)PX^{-1}(\sigma (s))|_0 \left| f\left( s,\varphi _s,\varphi ^\Delta _s\right) -f\left( s,\psi _s, \psi ^\Delta _s\right) \right| _0\Delta s\right. \\&\quad\left. +\int ^{+\infty }_{t}|X(t)(I-P)X^{-1}(\sigma (s))|_0\left| f\left( s,\varphi _s, \varphi ^\Delta _s\right) -f\left( s,\psi _s, \psi ^\Delta _s\right) \right| _0\Delta s\right| \\ &\le (L_1+L_2)\Vert \varphi -\psi \Vert _{\mathbb {X}}\left( \frac{k_1(1+\vartheta \alpha _1)}{\alpha _1} +\frac{k_2}{\alpha _2}\right) \end{aligned}$$

and

$$\begin{aligned} |(\Phi \varphi -\Phi \psi )^\Delta |_0 &= \left| \left[ \int _{-\infty }^tX(t)PX^{-1}(\sigma (s))\left( f\left( s,\varphi _s, \varphi ^\Delta _s\right) -f\left( s,\psi _s, \psi ^\Delta _s\right) \right) \Delta s\right. \right. \\&\quad\left. \left. -\int ^{+\infty }_{t}X(t)(I-P)X^{-1}(\sigma (s))\left( f\left( s,\varphi _s, \varphi ^\Delta _s\right) -f\left( s,\psi _s, \psi ^\Delta _s\right) \right) \Delta s\right] ^\Delta _t\right| _0\\ &= \left| f\left( t,\varphi _t, \varphi ^\Delta _t\right) -f\left( t,\psi _t, \psi ^\Delta _t\right) +A(t)\left[ \int _{-\infty }^tX(t)PX^{-1}(\sigma (s))\right. \right. \\&\quad\left. \left. \times \left( f\left( s,\varphi _s, \varphi ^\Delta _s\right) -f\left( s,\psi _s, \psi ^\Delta _s\right) \right) \Delta s\right. \right. \\&\quad\left. \left. -\int ^{+\infty }_{t}X(t)(I-P)X^{-1}(\sigma (s))\left( f\left( s,\varphi _s, \varphi ^\Delta _s\right) -f\left( s,\psi _s, \psi ^\Delta _s\right) \right) \Delta s\right] \right| _0\\ &\le (L_1+L_2)\left( ||A||\left( \frac{k_1(1+\vartheta \alpha _1)}{\alpha _1} +\frac{k_2}{\alpha _2}\right) +1\right) \Vert \varphi -\psi \Vert _{\mathbb {X}}. \end{aligned}$$

By $(H_3)$, we have $||\Phi \varphi -\Phi \psi ||_{\mathbb {X}}\le q\Vert \varphi -\psi \Vert _{\mathbb {X}}$. It follows that $\Phi$ is a contraction. Therefore, according to the Banach fixed-point theorem, $\Phi$ has a fixed point in ${\mathbb {X}}_0$, that is, (1) has a unique periodic solution in ${\mathbb {X}}_0$. This completes the proof of Theorem 1. $\square$

In view of Lemma 5 and Theorem 1, we have the following corollary:

### **Corollary 1**

*Let*$(H_1)$*and*$(H_3)$*hold. Suppose that*

$$(H_2')$$
*there is a constant*$\delta >0$*such that*
  $$|a_{ii}(t)|-\sum \limits _{j\ne i}|a_{ij}(t)|-\frac{1}{2}\mu (t)\left( \sum \limits _{j=1}^n|a_{ij}(t)|\right) ^2-\delta ^2\mu (t)\ge 2\delta ,\;t\in {\mathbb {T}},\quad i=1,2,\ldots ,n.$$

*Then* (1) *has a unique*$\omega$-*periodic solution*.

## Global exponential stability of periodic solution

### **Theorem 2**

*Let*$(H_1)$–$(H_3)$*hold. Suppose further that*

$$(H_4)$$
$$L_1+L_2+\frac{||A||}{N}<1$$*and*$$N(L_1+L_2)(1+\vartheta )||A||<\alpha$$.

*Then the periodic solution of* (1) *is globally exponentially stable*.

### *Proof*

By Theorem 1, (1) has an $\omega$-periodic solution *x*(*t*) with the initial value $\varphi (s)$. Suppose that *y*(*t*) is an arbitrary solution of (1) with the initial value $\psi (s)$. Denote $z(t)=y(t)-x(t)$. Then it follows from (1) that for $t\in {\mathbb {T}}$,

4 $$z^\Delta (t)=A(t)z(t)+f\left( t,y_t,y^\Delta _t\right) -f\left( t,x_t,x^\Delta _t\right) .$$

The initial value condition of (4) is

$$\phi (s)=\psi (s)-\varphi (s),\; \phi ^\Delta (s)=\psi ^\Delta (s)-\varphi ^\Delta (s),\; s\in [-\theta ,0]_{\mathbb {T}}.$$

By Lemma 1, for $t_0\in [-\theta ,0)_{\mathbb {T}}$ with $t_0<t$, we have

5 $$z(t) = e_ A(t,t_0)z(t_0)+\int _{t_0}^te_ A(t,\sigma (s))\left[ f\left( s,y_s,y^\Delta _s\right) -f\left( s,x_s,x^\Delta _s\right) \right] \Delta s.$$

Take a constant $0<\lambda <\alpha$ with $-\lambda \in {\mathcal {R}}^+$ and let

$$M>\max \left\{ \frac{N\alpha }{\alpha -N(L_1+L_2)(1+\vartheta )||A||}, \frac{N\alpha ||A||}{\alpha -(L_1+L_2)\left( \alpha +N(1+\vartheta )||A||^2\right) }\right\} .$$

By $(H_4)$, it is easy to verify that $M>1$ and hence, we have

$$||z||_{\mathbb {X}}\le M||\phi ||_{\mathbb {X}}e_{\ominus \lambda }(t,t_0),\quad \forall t\in [-\theta , t_0]_{{\mathbb {T}}}.$$

We claim that

6 $$||z||_{\mathbb {X}}\le M||\phi ||_{\mathbb {X}}e_{\ominus \lambda }(t,t_0),\quad \forall t\in (t_0,+\infty )_{{\mathbb {T}}}.$$

To prove this claim, we show that for any constant $p>1$, the following inequality holds

7 $$||z||_{\mathbb {X}}<p M||\phi ||_{\mathbb {X}}e_{\ominus \lambda }(t,t_0),\quad \forall t\in (t_0,+\infty )_{{\mathbb {T}}},$$

which means that

8 $$|z|_0<p M||\phi ||_{\mathbb {X}}e_{\ominus \lambda }(t,t_0),\quad \forall t\in (t_0,+\infty )_{{\mathbb {T}}}$$

and

9 $$|z^\Delta |_0<p M||\phi ||_{\mathbb {X}}e_{\ominus \lambda }(t,t_0),\quad \forall t\in (t_0,+\infty )_{{\mathbb {T}}}.$$

By way of contradiction, assume that (7) does not hold. We will have the following three cases. Case One: (9) is true and (8) is not true. Then there exists $t_1\in (t_0,+\infty )_{{\mathbb {T}}}$ such that

$$|z|_0\ge pM||\phi ||_{\mathbb {X}}e_{\ominus \lambda }(t_1,t_0),\;|z|_0< pM||\phi ||_{\mathbb {X}}e_{\ominus \lambda }(t,t_0),\quad t\in (t_0,t_1)_{{\mathbb {T}}}.$$

Hence, there must be a constant $c\ge 1$ such that

$$|z|_0=cp M||\phi ||_{\mathbb {X}}e_{\ominus \lambda }(t_1,t_0),\;|z|_0< pM||\phi ||_{\mathbb {X}}e_{\ominus \lambda }(t,t_0),\quad t\in (t_0,t_1)_{{\mathbb {T}}}.$$

Then, by (5), for $t=t_1$, we have

$$\begin{aligned} |z|_0 &= \left| e_ A(t,t_0)z(t_0)+\int _{t_0}^te_ A(t,\sigma (s))\left[ f\left( s,y_s,y^\Delta _s\right) -f\left( s,x_s,x^\Delta _s\right) \right] \Delta s\right| _0\\&\le |e_ A(t,t_0)|_0||\phi ||_{\mathbb {X}}+\left| \int _{t_0}^te_ A(t,\sigma (s))\left[ f\left( s,y_s,y^\Delta _s\right) -f\left( s,x_s,x^\Delta _s\right) \right] \Delta s\right| _0\\&\le |e_A(t,t_0)|_0||\phi ||_{\mathbb {X}} +\sup \limits _{t\in [0,\omega ]_{\mathbb {T}}}\left| \int _{t_0}^t|e_A(t,\sigma (s))|_0 |f\left( s,y_s,y^\Delta _s\right) -f\left( s,x_s, x^\Delta _s\right) |_0\Delta s\right| \\ &\le Ne_{-\alpha }(t_1,t_0)||\phi ||_{\mathbb {X}} +(L_1+L_2)||z||_{\mathbb {X}}\sup \limits _{t\in [0,\omega ]_{\mathbb {T}}}\left| \int _{t_0}^t|e_A(t,\sigma (s))|_0\Delta s\right| \\&\le Ne_{\ominus \lambda }(t_1,t_0)||\phi ||_{\mathbb {X}} +(L_1+L_2)c p Me_{\ominus \lambda }(t_1,t_0)\\&\quad\times\,||\phi ||_{\mathbb {X}} \sup \limits _{t\in [0,\omega ]_{\mathbb {T}}}\left| \int _{t_0}^t|(I+\mu (s)A(s))e_A(t,s)|_0\Delta s\right| \\&\le Ne_{\ominus \lambda }(t_1,t_0)||\phi ||_{\mathbb {X}} +(L_1+L_2)c p N Me_{\ominus \lambda }(t_1,t_0)\\&\quad\times\,||\phi ||_{\mathbb {X}}(1+\vartheta ||A||) \sup \limits _{t\in [0,\omega ]_{\mathbb {T}}}\left| \int _{t_0}^te_{-\alpha }(s,t_0)\Delta s\right| \\&\le Ne_{\ominus \lambda }(t_1,t_0)||\phi ||_{\mathbb {X}} +\frac{(L_1+L_2)c p N Me_{\ominus \lambda }(t_1,t_0)||\phi ||_{\mathbb {X}}(1+\vartheta )||A||}{\alpha }\\&< c p Me_{\ominus \lambda }(t_1,t_0)||\phi ||_{\mathbb {X}}\left( \frac{N}{M}+\frac{N(L_1+L_2) (1+\vartheta )||A||}{\alpha }\right) \\&< c p Me_{\ominus \lambda }(t_1,t_0)||\phi ||_{\mathbb {X}}, \end{aligned}$$

which is a contradiction.

Case Two: (8) is true and (9) is not true. Then there exists $t_2\in (t_0,+\infty )_{{\mathbb {T}}}$ such that

$$|z^\Delta |_0\ge pM||\phi ||_{\mathbb {X}}e_{\ominus \lambda }(t_2,t_0),\;|z^\Delta |_0< pM||\phi ||_{\mathbb {X}}e_{\ominus \lambda }(t,t_0),\quad t\in (t_0,t_2)_{{\mathbb {T}}}.$$

Hence, there must be a constant $b\ge 1$ such that

$$|z^\Delta |_0=bp M||\phi ||_{\mathbb {X}}e_{\ominus \lambda }(t_2,t_0),\;|z^\Delta |_0< pM||\phi ||_{\mathbb {X}}e_{\ominus \lambda }(t,t_0),\quad t\in (t_0,t_2)_{{\mathbb {T}}}.$$

In view of (5), for $t=t_2$, we have

$$\begin{aligned} |z^\Delta |_0 &= \left| A(t)e_ A(t,t_0)z(t_0)+ f\left( t,y_t, y^\Delta _t\right) -f\left( t,x_t, x^\Delta _t\right) \right. \\&\quad\left. +\,A(t)\int _{t_0}^te_A(t,\sigma (s))\left( f\left( s,y_s, y^\Delta _s\right) -f\left( s,x_s, x^\Delta _s\right) \right) \Delta s\right| _0\\ &\le ||A|||e_ A(t,t_0)|_0||\phi ||_{\mathbb {X}}+ \left| f\left( t,y_t, y^\Delta _t\right) -f\left( t,x_t, x^\Delta _t\right) \right| _0\\&\quad+\,\left| A(t)\int _{t_0}^te_A(t,\sigma (s))\left( f\left( s,y_s, y^\Delta _s\right) -f\left( s,x_s, x^\Delta _s\right) \right) \Delta s\right| _0\\&\le ||A||Ne_{-\alpha }(t_2,t_0)|\phi ||_{\mathbb {X}} +(L_1+L_2)b p Me_{\ominus \lambda }(t_2,t_0)|\phi ||_{\mathbb {X}}\\&\quad+\,\frac{||A||^2N b p M(L_1+L_2)e_{\ominus \lambda }(t_2,t_0)|\phi ||_{\mathbb {X}}(1+\vartheta )}{\alpha }\\&< b p Me_{\ominus \lambda }(t_2,t_0)|\phi ||_{\mathbb {X}}\left( \frac{N||A||}{M}+L_1+L_2+\frac{||A||^2N (L_1+L_2)(1+\vartheta )}{\alpha }\right) \\&< b p Me_{\ominus \lambda }(t_2,t_0)|\phi ||_{\mathbb {X}}, \end{aligned}$$

which is also a contradiction.

Case Three: (8) and (9) are both untrue. By Case One and Case Two, we can obtain a contradiction. Therefore, (7) holds. Let $p\rightarrow 1$, (6) holds. Hence, we have that

$$||y-x||_{\mathbb {X}}\le M||\varphi -\psi ||_{\mathbb {X}}e_{\ominus \lambda }(t,t_0),\quad t\in [-\theta ,\infty )_{{\mathbb {T}}},\; t\ge t_0,$$

which implies that the periodic solution *x*(*t*) of (1) is globally exponentially stable. This completes the proof of Theorem 2. $\square$

### **Corollary 2**

*Let*$(H_1)$, $(H_2')$*and*$(H_3)$–$(H_5)$*hold. Then* (1) *has a unique periodic solution, which is globally exponentially stable*.

## An example

In (1), if we take

$$\begin{aligned} A(t)=\left( \begin{array}{cccc} -0.002\sin 2t &{} 0\\ 0 &{} -0.001\cos 2t \end{array}\right) ,\quad f=(f_1,f_2)^T, \end{aligned}$$

where

$$\begin{aligned} f_1(t,\varphi ,\varphi ^\Delta ) &= 0.0003(\sin \varphi (-\tau (t))+\cos \varphi ^\Delta (-\zeta (t))),\\ f_2(t,\varphi ,\varphi ^\Delta )&= 0.0002(\sin \varphi (-\tau (t))+\cos \varphi ^\Delta (-\zeta (t))), \end{aligned}$$

$\tau , \zeta \in C({\mathbb {T}}, {\mathbb {T}}\cap {\mathbb {R}}^+)$ are $\pi$-periodic. Then (1) reduces to

10 $$\begin{aligned} \left( \begin{array}{lll} x^\Delta (t)\\ x^\Delta (t)\end{array}\right) &= \left( \begin{array}{ccc}-0.002\sin 2t &{} 0\nonumber \\ 0 &{} -0.001\cos 2t \end{array}\right) \left( \begin{array}{lll}x_1(t)\\ x_2(t)\end{array}\right) \nonumber \\&\quad +\left( \begin{array}{lll}0.0003(\sin \varphi (-\tau (t))+\cos \varphi ^\Delta (-\zeta (t)))\\ 0.0002(\sin \varphi (-\tau (t))+\cos \varphi ^\Delta (-\zeta (t)))\end{array}\right) . \end{aligned}$$

By a simple calculation, we have $L_1=L_2=0.0003$, $||A||=0.002$, $\ominus \alpha _1=-0.001$, $k_1=1$, $\ominus \alpha _2=k_2=0$, $\alpha =0.001$. One can easily verify that all the conditions in Corollary 2 are satisfied for $0\le \mu \le 1$. In particularly, if we take ${\mathbb {T}}={\mathbb {R}}$, then $\mu (t)=0$ and if we take ${\mathbb {T}}={\mathbb {Z}}$, then $\mu (t)=1$. Therefore, in both the cases of ${\mathbb {T}}={\mathbb {R}}$ and ${\mathbb {T}}={\mathbb {Z}}$, (10) has a $\pi$-periodic solution, which is exponentially stable.

### *Remark 4*

Example (10) shows that both the continuous case of (10)

$$\frac{{\mathrm {d}}}{{\mathrm {d}}t}x(t)=A(t)x(t)+f\left( t,x(t-\tau (t)),x'(t-\zeta (t))\right) ,\quad t\in {\mathbb {R}}$$

and its discrete analogue

$$\Delta x(n)=A(n)x(n)+f\left( n,x(n-\tau (n)),\Delta x(n-\zeta (n))\right) ,\quad n\in {\mathbb {Z}}$$

have the same dynamical property for the periodic case.

## Conclusion

In this paper, by using the existence of the exponential dichotomy of linear dynamic equations on time scales and the inequality techniques, we established the existence and global exponential stability of periodic solutions for a very general class of *n*-dimensional neutral dynamic equations on time scales. Our results of this paper are completely new and complementary to the previously known results even in both the case of differential equations (time scale ${\mathbb {T}}={\mathbb {R}}$) and the case of difference equations (time scale ${\mathbb {T}}={\mathbb {Z}}$), and our methods used in this paper may be used to study the problem of periodic solutions to other types of dynamic equations on time scales.
